# Reproduction and the pheromonal regulation of sex type in fern gametophytes

**DOI:** 10.3389/fpls.2015.00100

**Published:** 2015-03-06

**Authors:** Nadia M. Atallah, Jo Ann Banks

**Affiliations:** Department of Botany and Plant Pathology, Purdue University, West Lafayette, IN, USA

**Keywords:** antheridiogen, sex determination, ferns, GA signaling, GA biosynthesis

## Abstract

The fern life cycle includes a haploid gametophyte that is independent of the sporophyte and functions to produce the gametes. In homosporous ferns, the sex of the gametophyte is not fixed but can vary depending on its social environment. In many species, the sexual phenotype of the gametophyte is determined by the pheromone antheridiogen. Antheridiogen induces male development and is secreted by hermaphrodites once they become insensitive to its male-inducing effect. Recent genetic and biochemical studies of the antheridiogen response and sex-determination pathway in ferns, which are highlighted here, reveal many similarities and interesting differences to GA signaling and biosynthetic pathways in angiosperms.

## INTRODUCTION

**FIGURE 1:**
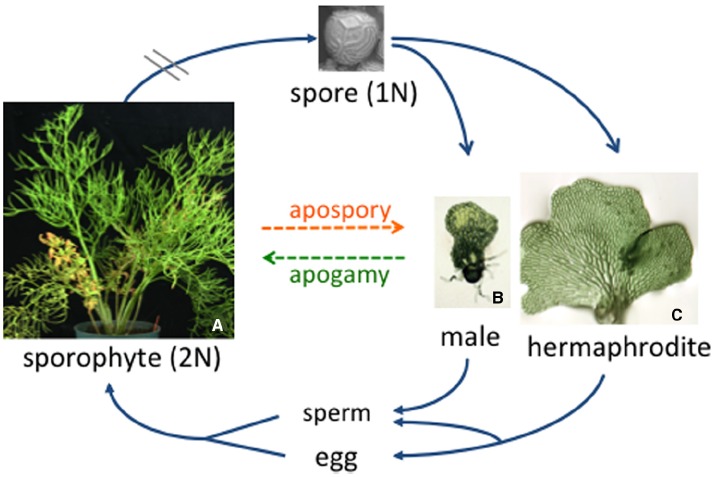
**The *C. richardii* life cycle.** Typical of all homosporous ferns, the diploid sporophyte produces sporangia on the abaxial surface of the fronds. Each sporangium contains haploid spores that are released from the sporophyte and, in the case of *C. richardii*, can remain dormant but viable for more than 50 years. Each spore germinates and develops as a male or hermaphroditic gametophyte depending on the presence or absence of antheridiogen. When mature, sperm are released and swim to the egg. The young sporophyte remains dependent on the gametophyte for a short period of time.

The fern life cycle, illustrated in Figure 1, features two distinct body types: the large diploid sporophyte and the tiny haploid gametophyte. From a reproduction point of view, the sole function of the sporophyte is to produce then release haploid spores, while the gametophyte, which grows from a spore, functions to produce the gametes. Some ferns, like all angiosperms, are heterosporous and produce both mega- and microspores that are destined to develop as female and male gametophytes, respectively. Most ferns species are homosporous and produce only one type of spore. While textbook drawings of homosporous fern gametophytes typically show a heart-shaped hermaphrodite, fern gametophytes can be male, female, male then female, female then male, hermaphroditic or asexual, depending on the species. In this review we highlight old and recent studies that have revealed the fascinating cross-talk that occurs between neighboring gametophytes in determining what their sexual phenotype will be.

## ASEXUAL REPRODUCTION IN FERN GAMETOPHYTES

In addition to reproducing sexually, there are many examples of fern gametophytes that circumvent sex and reproduce asexually. The most common type of asexual reproduction is apogamy, whereby a sporophyte plant develops from a gametophyte without fertilization, similar to apomixis in angiosperms. In naturally occurring apogamous species, the viable spores produced by the sporophyte have the same chromosome number as the sporophyte (Walker, 1962, 1979). Obligate apogamy often occurs naturally in species of ferns that produce no or only one type of gametangia. Because water is required for the flagellated sperm to swim to the egg in ferns, apogamous species are typically found in dry habitats where water is limiting (White, 1979). Apogamy also can be artificially induced in many ferns by adding sucrose to the culture media in which gametophytes are grown (Whittier and Steeves, 1962; White, 1979). By optimizing the conditions for inducing apospory in *Ceratopteris richardii* gametophytes, a recent study has established C. richardii as a useful experimental system for studying this phenomenon (Cordle et al., 2007). Induced apogamous sporophytes of *C. richardii* have features typical of the sporophyte, including stomata, vascular tissue and scale-like ramenta; however, they are abnormal compared to sexually-derived diploid sporophytes, which could be a consequence of being haploid. To better understand how sucrose promotes the development of a sporophyte from cells of the gametophyte, the same researchers identified 170 genes whose expression is up-regulated during the period of apogamy commitment. Many of them are associated with stress and metabolism or are homologs of genes preferentially expressed in seed and flower tissues (Cordle et al., 2012). Understanding apogamy, coupled with studies of apospory in *C. richardii*, where diploid gametophytes develop from cells of sporophyte leaves without meiosis (DeYoung et al., 1997), should provide useful insights into genes and molecular mechanisms that regulate the alternation of gametophyte and sporophyte generations in ferns in the absence of meiosis and fertilization.

A second form of asexual reproduction in homosporous ferns involves vegetative propagation of the gametophyte. While relatively rare, such gametophytes typically do not produce sex organs. The fern *Vittaria appalachiana*, for example, is only known from its gametophytes (Farrar and Mickel, 1991). Each gametophyte forms vegetative buds, or gammae, that allow gametophytes to multiply and form mats in dark, moist cavities and rock shelters in the Appalachian Mountains. While the origin of *V. appalachiana* (is it a recent hybrid or ancient relict?) and why it is unable to form sporophytes are unknown at this time, its persistent gametophyte suggest that fern gametophytes, like bryophyte gametophytes, can persist and thrive for very long periods of time.

## SEXUAL REPRODUCTION

Most homosporous ferns that reproduce sexually ultimately form hermaphroditic gametophytes that have antheridia and archegonia. While hermaphroditism increases the probability that a single gametophyte will reproduce, self-fertilization of a hermaphrodite (which is genetically similar to a doubled haploid in angiosperms) results in a completely homozygous sporophyte. Given that this absolute inbreeding could have negative consequences to the individual and reduce genetic variation in populations, it is not surprising that homosporous ferns have evolved mechanisms to promote outcrossing. One such mechanism that is common to many species of ferns involves the pheromonal regulation of sexual identity, where the sexual phenotype of an individual gametophyte depends on its social environment.

## ONE GENOTYPE—TWO OR MORE PHENOTYPES

In the late 1800’s, botanists began noting that fern gametophytes are often sexually dimorphic, with larger gametophytes bearing archegonia and smaller gametophytes bearing antheridia (Prantl, 1881; Yin and Quinn, 1995). The size difference between them was attributed to the presence or absence of a meristem, with females or hermaphrodites being “meristic” (with a meristem) and males “ameristic” (without a meristem). In a major discovery, Döpp noted that the medium harvested from cultures of *Pteridium aquilinum* gametophytes contained a pheromone that promoted the development of males in juvenile gametophytes (Döpp, 1950); this pheromone is referred to as antheridiogen. Antheridiogens or antheridiogen responses have since been identified in over 20 species of ferns (Yamane, 1998; Kurumatani et al., 2001; Jimenez et al., 2008).

**FIGURE 2:**
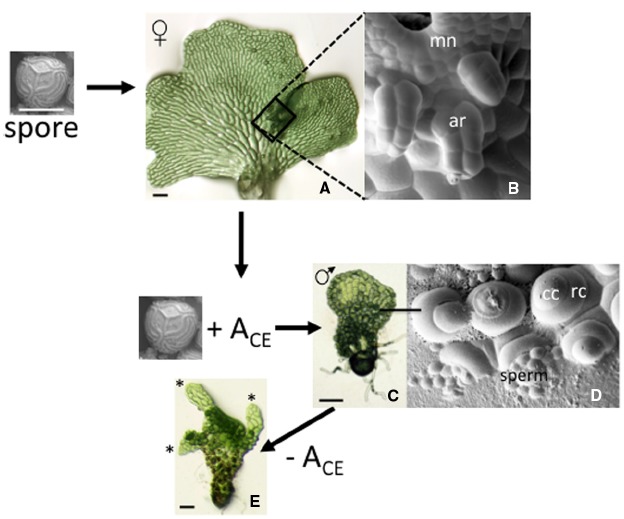
**The antheridiogen response in *C. richardii*.** A single spore always develops as a hermaphrodite when grown in the absence of A_CE_. The hermaphrodite consists of a single sheet of cells with a distinct multicellular meristem that forms a meristem notch and multiple archegonia that develop adjacent to the meristem notch, which are highlighted in the SEM (boxed area of the hermaphrodite). Hermaphrodites secrete A_CE_; in the presence of A_CE_, spores develop as males. The male lacks a meristem and almost all cells differentiate as antheridia. The SEM shows six antheridia, each having a ring cell and a cap cell that pops open to release sperm. When a male gametophyte is transferred to media lacking A_CE_, some cells divide and begin to form a hermaphroditic prothallus. The “switched” male shown is forming three such prothalli. mn: meristem notch; ar: archegonia; cc: cap cell; rc: ring cell.

Much of what is known about the biology of antheridiogen responses can be attributed to studies by Näf and Schraudolf during the 1950s and 1960s (reviewed in Näf, 1959, 1979). This response is illustrated here for the fern *C. richardii*, originally characterized by Hickok et al. (1995). In this species, an individual spore always develops as a relatively large hermaphrodite (Figure 2A) that produces egg-forming archegonia (Figure 2B), sperm-forming antheridia and multicellular lateral meristem. The hermaphrodite also secretes antheridiogen, or A_CE_ (for antheridiogen *Ceratopteris*) into its surroundings. If the hermaphrodite is removed then replaced with a genetically identical spore, the new spore will develop as an ameristic male gametophyte (Figure 2C) with many antheridia (Figure 2D) in response to A_CE_ secreted by the hermaphrodite. In a population of spores, spores that germinate first become hermaphrodites that secrete A_CE_, while slower-growing members of the population become male in response to the secreted A_CE_. In comparison to chromosomal based sex determination, this mechanism of sex-determination is unusual because it allows the ratio of males to hermaphrodites to vary depending on population size and density and it is inherently flexible rather than fixed.

Typical of other ferns, a *C. richardii* gametophyte is able to respond to A_CE_ for a limited period of time, prior to the establishment of a lateral meristem. The lateral meristem not only confers indeterminate growth to the gametophyte, but its formation coincides with a loss in ability to respond to A_CE_ as well as the secretion of A_CE_. Archegonia invariably initiate close to the meristem notch of the hermaphrodite, well after the lateral meristem is well developed. While the hermaphroditic program of expression cannot be reversed, the male program of expression is reversible. Cells of the male gametophyte prothallus, when transferred to media lacking A_CE_, will divide to ultimately form one or more new hermaphroditic prothalli (Figure 2E). Antheridiogen thus serves multiple functions in male gametophyte development: it represses divisions of the prothallus that establish the lateral meristem; it promotes the rapid differentiation of antheridia; it represses its own biosynthesis; and it serves to maintain in the gametophyte an ability to respond to itself.

All of the antheridiogens that have been structurally characterized from ferns are gibberellins (GAs) (Yamane et al., 1979; Furber et al., 1989; Takeno et al., 1989; Yamane, 1998). Although the structure of ACE is unknown, GA biosynthetic inhibitors reduce the proportion of males in a population of *C. richardii* gametophytes suggesting that ACE and GA share a common biosynthetic pathway (Warne and Hickok, 1989). ABA, a known antagonist of GA responses in angiosperms, completely blocks the ACE response in *C. richardii*, also indicating that ACE is likely a GA (Hickok, 1983).

## THE SEX-DETERMINING PATHWAY IN *Ceratopteris*

**FIGURE 3:**
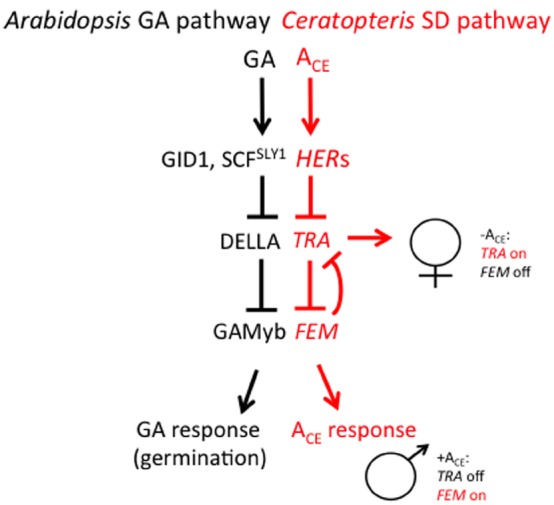
**A comparison of the GA signaling pathway in angiosperms and the sex-determining (SD) pathway in *C. richardii.*** The SD pathway in *C. richardii* is based solely on the epistatic interactions among sex-determining mutants but it is consistent with recent molecular and biochemical studies in the fern *L. japonicum*. T bars represent repressive events whereas arrows indicate activating events.

Most recent studies aimed at understanding how antheridiogen determines the sex of the gametophyte have focused on two species of homosporous ferns: *C. richardii* and *Lygodium japonicum*. *Ceratopteris richardii* is a semi-tropical, annual species and is useful as a genetic system for many reasons. Large numbers of single-celled, haploid spores (typically 10^6^) can be mutagenized and mutants identified within 2 weeks after mutagenesis. Gametophytes can be dissected and regrown, making it possible to simultaneously self-fertilize and out-cross a single mutant gametophyte. Because self-fertilization of a gametophyte results in a completely homozygous sporophyte that produces >10^7^ spores within a 6-month period, suppressor mutants are also easy to generate. Because *C. richardii* gametophytes are sexually dimorphic, mutations affecting the sex of the gametophyte are especially easy to identify (Hickok, 1977, 1985; Hickok et al., 1985, 1991; Warne and Hickok, 1986; Warne et al., 1988; Hickok and Schwarz, 1989; Vaughn et al., 1990; Scott and Hickok, 1991; Chun and Hickok, 1992; Banks, 1994; 1997a,b; Eberle and Banks, 1996; Strain et al., 2001; Renzaglia et al., 2004). Over 70 mutants affecting sex determination have been characterized, most falling into three major phenotypic groups: the *hermaphroditic* (*her*) mutants, which are hermaphroditic in the presence or absence of A_CE_, the *transformer* (*tra*) mutants, which are male in the presence or absence of A_CE_, and the *feminization* (*fem*) mutants, which are female in the presence or absence of A_CE_ and produce no antheridia. Through test of epistasis (i.e., comparing mutant phenotypes of single and various combinations of double and triple mutants), a genetic model of the sex determination pathway has been developed and is illustrated in Figure 3 (Eberle and Banks, 1996; Banks, 1997a,b; Strain et al., 2001). This pathway reveals that there are two major regulators of sex: *TRA*, which is necessary for lateral meristem and archegonia development (female traits), and *FEM*, which is necessary for antheridia development (the male trait). *FEM* and *TRA* negatively regulate each other such that only one can be expressed in the gametophyte. What determines whether *FEM* or *TRA* is expressed in the gametophyte is A_CE_. A_CE_ activates the *HERs*, which, in turn, repress *TRA*. Because *TRA* cannot repress *FEM*, *FEM* is expressed and the gametophyte develops as a male. In the absence of A_CE_, *HER* is not active and is thus unable to repress *TRA*. *TRA* promotes the development of a gametophyte with female traits and represses the development of antheridia by repressing the *FEM* gene that promotes male development. Additional genetic experiments have revealed that the repression of *FEM* by *TRA* and of *TRA* by *FEM* is indirect and involves other genes (Strain et al., 2001). What is remarkable about this pathway is that it is inherently flexible, which is consistent with what is understood about sex determination in this species by A_CE_. This “battle of the sexes”—deciding whether to be male or female—depends on which of the two major regulatory sex genes prevails in the young gametophyte, a decision that is ultimately determined by the presence or absence A_CE_.

While this model explains how male and female gametophyte identities are determined, it does not explain the hermaphrodite. One possibility is that in certain cells of the hermaphrodite, the activities of *FEM* and *TRA* are reversed, allowing *FEM* to be expressed in cells that will eventually differentiate as antheridia. Testing this and other possibilities will require the cloning of the sex-determining genes and assessing their temporal and spatial patterns of expression in the developing hermaphrodite.

The sex-determining pathway in *C. richardii* is remarkable in its resemblance to the GA signaling pathway in angiosperms (Sun, 2011), as illustrated in Figure 3. In *Arabidopsis*, GA is bound by its receptor GIBBERELLIN INSENSITIVE DWARF1 (GID1). The GA-GID1 complex triggers the rapid proteolysis of one or more DELLA proteins that are ultimately responsible for repressing GA responses. Proteolysis of DELLA requires GID1 and the specific F-box protein SLEEPY1 (SLY1), which promotes poly-ubiquitination of DELLA by the SCR^SLY1/GID2^ complex and results in its degradation by the 26S proteasome. Since DELLA acts as a repressor of GA responses, its GA-induced degradation results in a GA response. While targets of DELLA repression have been identified (Fleet and Sun, 2005), in the case of barley seed germination (which requires GA), DELLA directly or indirectly represses *GAMYB*, a transcription factor that promotes α-amylase expression in germinating barley seeds (Gubler et al., 1995, 1999). Based on the similarities between the GA signaling pathway in angiosperms and the sex determination pathway in *C. richardii*, it is tempting to speculate that the *HER* genes in *C. richardii* encode GID1 and SLY1, that *TRA* encodes a DELLA protein, and that *FEM* encodes a GAMYB-like protein.

## ANTHERIDIOGEN BIOSYNTHESIS IS SPLIT BETWEEN YOUNG AND OLDER GAMETOPHYTES IN *Lygodium japonicum*

*Lygodium japonicum* is another homosporous fern species with an antheridiogen response. This species has the distinct advantage of having its antheridiogens structurally well characterized. Two different GAs have been identified as antheridiogens in this species, including GA_9_ methyl ester (Yamane et al., 1979) and GA_73_ methyl ester (Yamane et al., 1988). GA_73_ methyl ester is the most active antheridiogen and is able to induce antheridia formation at the incredibly low concentration of 10^–15^ M. To test the hypothesis that antheridiogen is synthesized through the GA biosynthetic pathway, *L. japonicum genes* related to five different GA synthesis genes, including *ent-copalyl diphosphate/ent-kaurene synthase* (*CPS/KS*), *ent-kaurenoic acid oxidase* (*KAO*), *kaurene oxidase* (*KO*), *GA 20-oxidase* (*GA20ox*), and *GA3-oxidase* (*GA3ox*), were identified and their expression patterns in developing gametophytes investigated (Tanaka et al., 2014). Their expression patterns revealed that all but *GA30ox* were more highly expressed in older gametophytes that secrete antheridiogen, consistent with the expectation that antheridiogen biosynthesis genes are up-regulated in gametophytes that secrete it. *GA3ox* expression showed the opposite pattern of expression; i.e., it was more highly expressed in young gametophytes that did not secrete antheridiogen but were capable of responding to antheridiogen. To explore this further, the same authors assayed the effects of prohexadione, a GA3ox inhibitor, on antheridia formation in the presence of GA_4_ (which has an OH group at the C3 position) or GA_9_ methyl ester (which lacks the OH group at C3); both GA_9_ and GA_4_ induce antheridia formation by themselves. Whereas prohexadione plus GA_9_ methyl ester inhibited antheridia formation, prohexadione plus GA_4_ did not, demonstrating that C3 hydroxylation of antheridiogen is essential for inducing antheridia formation. In another series of experiments, the authors found that GA_9_ methyl ester was converted to GA_9_ in young gametophytes. Based on these and other results, a model was proposed whereby antheridiogen (GA_9_ methyl ester) is synthesized via a GA biosynthetic pathway and secreted by older gametophytes. When it is taken up by younger gametophytes, the methyl ester is removed by a possible methyl esterase then hydroxylated at the C3 position by GA3ox to GA_4_, where it is perceived and transduced by the GA signaling pathway in young gametophyte. Because GA_9_ methyl ester is more hydrophobic and more efficiently taken up by gametophytes than GA_9_, splitting the GA biosynthetic pathway between young and older gametophytes was proposed to enhance the sensitivity of young gametophytes to the secreted antheridiogen by their neighbors and, at the same time, promote the activation of male traits once inside the young gametophyte (Tanaka et al., 2014).

In addition to characterizing antheridiogen biosynthesis in *L. japonicum*, Tanaka et al. (2014) also made two other important discoveries. They found that a *L. japonicum* DELLA protein was degraded in GA_4_ and GA_9_ methyl ester treated gametophytes, and that the *L. japonicum* GID1 and DELLA proteins could interact in a yeast–two hybrid assay, but only in the presence of GA_4_ (and not GA_4_ methyl ester or GA_9_ methyl ester). All told, the results of these experiments were used to define a model of the antheridiogen response in *L. japonicum* that is remarkably similar to the pathways illustrated in Figure 3.

## FUTURE DIRECTIONS

The elucidation of the antheridiogen biosynthetic and signaling pathways in ferns has only just begun and many questions regarding sex determination and sexual reproduction remain, many of which can be resolved by cloning all of the sex determining genes. Some of these questions are: To what extent are other hormones involved in sex determination? Is the split GA biosynthetic pathway in *L. japonicum* typical of other ferns? What is the relationship between the antheridiogen response in the gametophyte to GA responses in the sporophyte? Knowing that some mutations in *C. richardii* (e.g., *her* mutations) have no effect on the sporophyte while other mutations (e.g., *tra* mutations) severely affect the sporophyte suggest that at least some, but not all, genes are necessary in both generations. Is antheridiogen also involved in the developmental decision to produce mega- and micro-sporangia in heterosporous ferns? From an evolutionary perspective, was the antheridiogen signaling and responses in the gametophyte co-opted during or important for the evolution of heterospory from homospory in ferns? Addressing these and other questions will lead to a more comprehensive understanding of sex determination in ferns, including an understanding of the molecular mechanisms at play.

### Conflict of Interest Statement

The authors declare that the research was conducted in the absence of any commercial or financial relationships that could be construed as a potential conflict of interest.
